# Predictors of post-thrombolysis symptomatic intracranial hemorrhage in Chinese patients with acute ischemic stroke

**DOI:** 10.1371/journal.pone.0184646

**Published:** 2017-09-18

**Authors:** Mingyong Liu, Yuesong Pan, Lichun Zhou, Yongjun Wang

**Affiliations:** 1 Center of Stroke, Beijing Tiantan Hospital, Capital Medical University, Beijing, China; 2 National Clinical Research Center for Neurological Diseases, Beijing, China; 3 Center of Stroke, Beijing Institute for Brain Disorders, Beijing, China; 4 Beijing Key Laboratory of Translational Medicine for Cerebrovascular Disease, Beijing, China; 5 Department of Neurology, Beijing Chaoyang Hospital, Capital Medical University, Beijing, China; 6 Department of Epidemiology and Health Statistics, School of Public Health, Capital Medical University, Beijing, China; 7 Beijing Municipal Key Laboratory of Clinical Epidemiology, Beijing, China; Massachusetts General Hospital, UNITED STATES

## Abstract

**Background and purpose:**

Predictors of symptomatic intracranial hemorrhage (sICH) in Chinese patients with acute ischemic stroke treated with recombinant tissue plasminogen activator remain unclear.

**Methods:**

Data from the Thrombolysis Implementation and Monitor of Acute Ischemic Stroke in China (TIMS-China) study were assessed to explore risk factors for symptomatic intracranial hemorrhage after intravenous thrombolysis. Three candidate sICH definitions were analyzed.

**Results:**

Among 1128 patients with acute ischemic stroke treated with intravenous rtPA within 4.5 hours of symptom onset, 23 (2.0%), 44(3.9%) and 61 (5.4%) experienced modified mSITS-MOST, ECASS II, and NINDS defined sICH, respectively. Multivariate logistic regression revealed independent risk factors for sICH were age≧70 years-old(sICH per NINDS, adjusted OR = 1.73[95%CI1.02–2.95],*p* = 0.04),diabetes(sICH per SITS-MOST, adjusted OR = 3.50 [95%CI1.34–9.16], *p* = 0.01), serum glucose on admission >9.0mmol/L(sICH per ECASS II, adjusted OR = 2.84[95%CI1.48–5.46], *p* = 0.002),NIHSS on admission>20(sICH per SITS-MOST, adjusted OR = 5.06[95%CI1.68–15.20], *p* = 0.004 or sICH per NINDS, adjusted OR 2.81[95%CI1.42–5.57], p = 0.003) and cardioembolism(sICH per SITS-MOST, adjusted OR = 7.09[95%CI2.41–20.87], *p*<0.001 or sICH per ECASS II, adjusted OR = 4.99[95%CI2.53–9.84], p<0.001)or sICH per NINDS, adjusted OR = 2.47[95%CI1.39–4.39], *p* = 0.002).

**Conclusion:**

Cardioembolism, NIHSS on admission higher than 20, serum glucose on admission higher than 9.0 mmol/L and age ≧70 years were independent risk factors for symptomatic intracranial hemorrhage in Chinese patients with acute ischemic stroke treated with recombinant tissue plasminogen activator.

## Introduction

Recombinant tissue plasminogen activator(r-tPA) treatment is an effective therapy for acute ischemic stroke(AIS)[1]. However, treatment with r-tPA is accompanied by a fatal complication known as symptomatic intracranial hemorrhage (sICH), which can worsen the outcomes of stroke patients[2].According to NINDS criteria, the incidence rate of sICH is 2.2% to 8% across the world[3–7], and 4.87% to 7.3% in China[8–9].Ginsberg reported that symptomatic intracranial hemorrhage is fatal in 41.4% of cases [10]. In a survey of American emergency physicians, 26% of the 1105 respondents were reluctant to use thrombolysis in acute ischemic stroke for fear of sICH[11]. Therefore, it is critical to identify predictors of sICH after thrombolysis. As demonstrated in the GRASPS score, Asian race was an independent predictor of sICH[12]. It has been postulated that Asian had higher rates of tPA-related intracranial hemorrhage because of the racial differences in blood coagulation–fibrinolysis factors, such as in the altered functions of fibrinogen and factor XII[13]. So,we think it is worthwhile to investigate the potential predictors in the Chinese population. In China, there is limited information regarding risk factors for sICH in patients with acute ischemic stroke treated with r-tPA. The aim of this study was to identify independent risk factors associated with sICH in Chinese patients with acute ischemic stroke treated with r-tPA.

## Methods

### Study population

Data were derived from a Chinese national prospective stroke registry study—The Thrombolysis Implementation and Monitor of Acute Ischemic Stroke in China (TIMS-China) trial. The design and previous results of the TIMS-China registry were published elsewhere[14]. Briefly, consecutive patients (18–80 years-old; platelet≧100,000/mm^3^) who received intravenous r-tPA(Actilyse; Boehringer Ingelheim,Germany) within 4.5 hours of AIS onset were recruited from 67 hospitals in China, between May 2007 and April 2012[14]. The TIMS-China was approved by the ethics committees at all participating hospitals. Written informed consent was obtained from all patients or their representatives before data collection.

This secondary analysis of data from the TIMS-China registry study was conducted from March 2016 to June 2016.

### Data collection

Data on patient demographics, clinical characteristics, computed tomography (CT) or magnetic resonance imaging (MRI) scans of the brain, medical therapy and IV thrombolysis were collected by face-to-face interview by neurologists from the participating hospitals.

Potential risk factors included age, gender, hypertension, diabetes, modified Rankin Scale(mRS) before stroke, NIHSS on admission, SBP on admission, DBP on admission, Serum glucose on admission, pre-existing treatment, early infarct signs on admission CT head scan, hyper dense middle cerebral artery sign on admission CT head scan, alteplase dose (mg/kg body weight),onset to needle time (hours),onset to needle time >3 hours, and stroke subtype.

### Outcome measurements

The primary outcome was incidence of sICH at 0 to 36 h after IV r-tPA. Symptomatic ICH (sICH) was based on three different definitions from previously published international trials: parenchymal hemorrhage with deterioration in National Institutes of Health Stroke Scale score of≧4 points or death (the modified Safe Implementation of Thrombolysis in Stroke-Monitoring Study [mSITS-MOST])[15]; any type of intracranial hemorrhage on any post-treatment imaging after thrombolysis start and increase of NIHSS by 4points from baseline, or death(the European Cooperative Acute Stroke Study [ECASS] II)[16]; any hemorrhagic transformation temporally related to any worsening in neurological condition (National Institute of Neurological Disorders and Stroke [NINDS])[17].

### Statistical analysis

Continuous and categorical variables of patients’ baseline characteristics were presented as mean±SD or median (interquartile range) and percentages, respectively. Baseline characteristics of patients with and without sICH per SITS-MOST, ECASS II and NINDS definitions were compared by the Mann-Whitney U test and Pearson χ^2^ method for continuous and categorical variables, respectively. Odds ratios (ORs) with 95% confidence intervals (CIs) were calculated by multi-variable logistic regression analysis. Two-sided *p*<0.05 was considered statistically significant. All analyses were performed with the SAS 9.4 software.

## Results

From May 2007 to April 2012, 1128 AIS patients with onset to needle time ≦4.5 hours were entered into the TIMS-China registry. Among the 1128 patients with acute ischemic stroke included in this study, 23 (2.0%), 44 (3.9%), and 61 (5.4%) experienced modified mSITS-MOST, ECASS II, and NINDS defined sICH, respectively.

Demographic and baseline characteristics of the patients with sICH per mSITS-MOST criteria are summarized in Table 1.Compared with non-sICH patients, sICH patients were accompanied with higher NIHSS on admission(18 [12–21] vs11 [7–16],*p* = 0.0007), higher serum glucose on admission(8.64±3.04vs7.70±3.02mmol/L,*p* = 0.0410),and more cardioembolism stroke subtype (13 [56.52%] vs 208 [18.82%],*p*<0.0001).

**Table 1:** Demographic and baseline characteristics of the patients with sICH per mSITS-MOST criteria.

variables	sICH (N = 23)	Non-sICH(N = 1105)	p
Age (year)	66.22±8.89	63.43±11.38	0.3640
<70	12(52.17)	699(63.26)	0.2757
≧70	11(47.83)	406(36.74)	
Gender (male)	12(52.17)	676(61.18)	0.3810
Hypertension	15(65.22)	652(59.00)	0.5486
Diabetes	7(30.43)	189(17.10)	0.1639
mRS 0–2 before stroke	23(100.00)	1083(98.01)	0.4944
NIHSS on admission	18(12–21)	11(7–16)	0.0007
0–14	8(34.78)	762(68.96)	0.0006
15–20	8(34.78)	233(21.09)	
>20	7(30.43)	110(9.95)	
SBP on admission (mm Hg)	148.04±15.06	148.03±21.06	0.9463
DBP on admission (mm Hg)	86.00±11.78	85.94±12.67	0.9003
Serum glucose on admission (mmol/L)	8.64±3.04	7.70±3.02	0.0410
≦9.0	14(60.87)	894(80.90)	0.0328
>9.0	9(39.13)	211(19.10)	
Preexisting antihypertensive treatment	7(30.43)	425(38.46)	0.4332
Preexisting anticoagulant treatment	0(0.00)	19(1.72)	0.5259
Preexisting antiplatelet treatment	6(26.09)	152(13.76)	0.1667
Early infarct signs on admission CT head scan	0(0.00)	61(5.52)	0.2466
Hyper dense middle cerebral artery sign on admission CT	2(8.70)	75(6.79)	1.0000
Alteplase dose (mg/kg body weight)	0.90(0.81–0.90)	0.90(0.86–0.90)	0.2460
Onset to needle time (hours)	3.03(2.38–3.58)	2.83(2.33–3.25)	0.2553
Onset to needle time >3 h	12(52.17)	362(32.76)	0.0503
Stroke subtype			
Large-artery atherosclerosis	5(21.74)	601(54.39)	<.0001
Small-vessel occlusion	0(0.00)	117(10.59)	
Cardioembolism	13(56.52)	208(18.82)	
Other	5(21.74)	179(16.20)	

Continuous variables: median with interquartile range (IQR) and P values per the Mann-Whitney U test. Categorical variables: proportions (%) and P values per the Pearson χ^2^ test.

The sICH indicates symptomatic intracranial hemorrhage; SITS-MOST, Safe Implementation of Thrombolysis in Stroke-Monitoring Study; NIHSS, National Institutes of Health Stroke Scale; BP, blood pressure; mRS, modified Rankin Scale

Demographic and baseline characteristics of the patients with sICH per ECASS II criteria are summarized in Table 2.Compared with non-sICH patients, sICH patients were accompanied with higher NIHSS on admission(15 [12–20] vs11 [7–16], *p* = 0.0002),higher serum glucose on admission(8.88±3.81vs7.67±2.99mmol/L,*p* = 0.0150),and more cardioembolism stroke subtype (23 [52.27%] vs 198 [18.27%], *p*<0.0001).

**Table 2:** Demographic and baseline characteristics of the patients by sICH per ECASS II criteria.

variables	sICH (N = 44)	Non-sICH(N = 1084)	p
Age (year)	66.73±9.04	63.35±11.41	0.0918
<70	22(50.00)	689(63.56)	0.0677
≧70	22(50.00)	395(36.44)	
Gender (male)	24(54.55)	664(61.25)	0.3711
Hypertension	28(63.64)	639(58.95)	0.5352
Diabetes	9(20.45)	187(17.25)	0.5825
mRS 0–2 before stroke	44(100.00)	1062(97.97)	0.3399
NIHSS on admission	15(12–20.00)	11(7–16)	0.0002
0–14	21(47.73)	749(69.10)	0.0041
15–20	13(29.55)	228(21.03)	
>20	10(22.73)	107(9.87)	
SBP on admission (mm Hg)	146.48±17.92	148.09±21.07	0.5893
DBP on admission (mm Hg)	87.00±11.56	85.90±12.69	0.7532
Serum glucose on admission (mmol/L)	8.88±3.81	7.67±2.99	0.0150
≦9.0	28(63.64)	880(81.18)	0.0040
>9.0	16(36.36)	204(18.82)	
Preexisting antihypertensive treatment	17(38.64)	415(38.28)	0.9624
Preexisting anticoagulant treatment	0(0.00)	19(1.75)	0.3758
Preexisting antiplatelet treatment	10(22.73)	148(13.65)	0.0891
Early infarct signs on admission CT head scan	2(4.55)	59(5.44)	1.0000
Hyper dense middle cerebral artery sign on admission CT	5(11.36)	72(6.64)	0.3615
Alteplase dose (mg/kg body weight)	0.90(0.80–0.90)	0.90(0.86–0.90)	0.0482
Onset to needle time (hours)	2.88(2.41–3.55)	2.83(2.33–3.25)	0.2754
Onset to needle time >3 h	20(45.45)	354(32.66)	0.0771
Stroke subtype			
Large-artery atherosclerosis	15(34.09)	591(54.52)	<.0001
Small-vessel occlusion	0(0.00)	117(10.79)	
Cardioembolism	23(52.27)	198(18.27)	
Other	6(13.64)	178(16.42)	

Continuous variables: median with interquartile range (IQR) and P values per the Mann-Whitney U test. Categorical variables: proportions (%) and P values per the Pearson χ^2^ test.

age; ECASS II, the European Cooperative Acute Strokage; ECASS II, the European Cooperative Acute Stroke Study II; NIHSS, National Institutes of Health Stroke Scale; BP, blood pressure; mRS, modified Rankin Scale

Demographic and baseline characteristics of the patients by sICH per NINDS criteria are summarized in Table 3.Compared with non-sICH patients, sICH patients were accompanied with elder age (67.18±9.28vs 63.27±11.42,*p* = 0.0126), higher NIHSS on admission(16 [12–20] vs11 [7–16],*p* = 0.0001), larger proportion of serum glucose >9.0 mmol/L on admission(18 [29.51%] vs202 [18.93%],*p* = 0.0426),and more cardioembolism stroke subtype (27 [44.26%] vs194 [18.18%], *p*<0.0001).

**Table 3:** Demographic and baseline characteristics of the patients by sICH per NINDS criteria.

variables	sICH (N = 61)	Non-sICH(N = 1067)	p
Age (year)	67.18±9.28	63.27±11.42	0.0126
<70	28(45.90)	683(64.01)	0.0044
≧70	33(54.10)	384(35.99)	
Gender (male)	32(52.46)	656(61.48)	0.1600
Hypertension	37(60.66)	630(59.04)	0.8033
Diabetes	10(16.39)	186(17.43)	0.8351
mRS 0–2 before stroke	61(100.00)	1045(97.94)	0.2574
NIHSS on admission	16(12–20)	11(7–16)	<.0001
0–14	27(44.26)	743(69.63)	<.0001
15–20	19(31.15)	222(20.81)	
>20	15(24.59)	102(9.56)	
SBP on admission (mm Hg)	148.10±18.21	148.03±21.11	0.9850
DBP on admission (mm Hg)	87.07±10.99	85.88±12.73	0.5413
Serum glucose on admission (mmol/L)	8.35±3.47	7.68±3.00	0.1114
≦9.0	43(70.49)	865(81.07)	0.0426
>9.0	18(29.51)	202(18.93)	
Preexisting antihypertensive treatment	22(36.07)	410(38.43)	0.7123
Preexisting anticoagulant treatment	0(0.00)	19(1.78)	0.2932
Preexisting antiplatelet treatment	11(18.03)	147(13.78)	0.3516
Early infarct signs on admission CT head scan	3(4.92)	58(5.44)	1.0000
Hyper dense middle cerebral artery sign on admission CT	8(13.11)	69(6.47)	0.0816
Alteplase dose (mg/kg body weight)	0.90(0.83–0.90)	0.90(0.86–0.90)	0.3226
Onset to needle time (hours)	2.75(2.38–3.33)	2.83(2.33–3.25)	0.8085
Onset to needle time >3 h	21(34.43)	353(33.08)	0.8285
Stroke subtype			
Large-artery atherosclerosis	27(44.26)	579(54.26)	<.0001
Small-vessel occlusion	0(0.00)	117(10.97)	
Cardioembolism	27(44.26)	194(18.18)	
Other	7(11.48)	177(16.59)	

Continuous variables: median with inter quartile range (IQR) and P values per the Mann-Whitney U test. Categorical variables: proportions (%) and P values per the Pearson χ^2^ test.

The sICH indicates symptomatic intracranial hemorrhage; NINDS, the National Institute of Neurological Disorders and Stroke; NIHSS, National Institutes of Health Stroke Scale; BP, blood pressure; mRS, modified Rankin Scale

Multivariate logistic regression analysis for determining sICH risk factors per mSITS-MOST,ECASS II or NINDS are summarized in Table 4. Independent risk factors for sICH were age≧70 years (sICH per NINDS, adjusted OR = 1.73 [95%CI 1.02–2.95],*p* = 0.04),diabetes(sICH per SITS-MOST, adjusted OR = 3.50 [95%CI1.34–9.16], *p* = 0.01), serum glucose on admission >9.0mmol/L(sICH per ECASS II, adjusted OR = 2.84 [95%CI1.48–5.46],*p* = 0.002),NIHSS on admission>20(sICH per SITS-MOST, adjusted OR = 5.06 [95%CI1.68–15.20],*p* = 0.004 or sICH per NINDS, adjusted OR = 2.81 [95%CI1.42–5.57],p = 0.003) and cardioembolism (sICH per SITS-MOST, adjusted OR = 7.09 [95%CI2.41–20.87],*p*<0.001 or sICH per ECASS II, adjusted OR = 4.99 [95%CI2.53–9.84],p<0.001 or sICH per NINDS, adjusted OR2.47 [95%CI1.39–4.39],*p* = 0.002).

**Table 4:** Results of multivariate logistic regression analysis of sICH risk factors per mSITS-MOST,ECASS II or NINDS.

	sICH per mSITS-MOST		sICH per ECASS II		sICH per NINDS	
	Adjusted OR(95%CI)	p	Adjusted OR(95%CI)	p	Adjusted OR(95%CI)	p
Age≧70year					1.73(1.02–2.95)	0.04
Diabetes	3.50(1.34–9.16)	0.01				
Serum glucose on admission >9.0mmol/L			2.84(1.48–5.46)	0.002		
NIHSS on admission						
0–14	ref				ref	
15–20	2.37(0.83–6.76)	0.11			1.54(0.82–2.90)	0.18
>20	5.06(1.68–15.20)	0.004			2.81(1.42–5.57)	0.003
Stroke subtype						
Large-artery atherosclerosis	ref		ref		ref	
Small-vessel occlusion	NA	-	NA	-	NA	-
Cardioembolism	7.09(2.41–20.87)	<0.001	4.99(2.53–9.84)	<0.001	2.47(1.39–4.39)	0.002
Other	5.12(1.40–18.72)	0.01	1.46(0.56–3.85)	0.44	0.99(0.42–2.32)	0.97

Multivariate logistic regression analysis adopted backward method. Ref, reference; N/A, not available or unreported

The sICH indicates symptomatic intracranial hemorrhage; SITS-MOST, Safe Implementation of Thrombolysis in Stroke-Monitoring Study; ECASS II, the European Cooperative Acute Stroke Study II; NINDS, the National Institute of Neurological Disorders and Stroke; NIHSS, National Institutes of Health Stroke Scale; BP, blood pressure; mRS, modified Rankin Scale

## Discussion

This study showed that age≧70 years, NIHSS score>20, diabetes or serum glucose on admission >9.0mmol/L and cardioembolism were independent predictors of sICH after thrombolysis in Chinese patients with acute ischemic stroke.

Because age> 80 years old is considered a relative contraindication for 3–4.5h iv r-tPA thrombosis by many guidelines, and the time range in this study was 0h-4.5h, we selected patients with age varying from 18 to 80 years. It was reported that higher sICH are found in patients ≧70 years old treated by iv r-tpA.[18] Therefore, age≧70 years was considered a risk factor for sICH. In agreement with our findings, Sombat M, et al reported that age>75 years increasesby1.532 times the risk of sICH in Thai AIS patients who accepted iv r-tPA thrombosis[19].However, two studies in Australia[20] and Japan[21] reported that age over 80 years is not related to sICH risk increase. These findings indicated that ethnicity may account for the discrepant effects of age in sICH.

That higher NIHSS score increases the risk of sICH has been reported by many studies[2,19,22].Severe ischemic stroke is reflected by large areas of injured brain tissue, including injured blood vessels, which are prone to bleeding after r-tPA treatment.

Lindley RI et al. reported diabetes does not increase the risk of sICH in AIS patients treated with r-tPA[23]. However, Menon BK et al reported that serum glucose on admission >8.3mmol/L increases sICH risk[12], corroborating the above findings. Certainly, the mechanism behind this phenomenon requires further research.

Cardioembolism was reported as an independent predictor of sICH in many studies[5,24–27]. This was confirmed in the present study assessing Chinese patients. Mosimann PJ et al reported that onset to needle time >3h increases the risk of sICH[28]. We also found such a trend, although no statistical significance was reached (52.17% vs 32.76%, *p* = 0.0503).

In agreement with a previous study[29], we demonstrated that pre-existing anticoagulant treatment did not increase the risk of sICH (0.00% vs1.72%,*p* = 0.5259). Matute MC et al. reported that preexisting oral anticoagulant treatment with INR <2 does not increase the risk of sICH. However, prior use of low molecular heparin appeared to increase the risk of sICH[30]. Regarding bleeding effects of anticoagulants before thrombolysis, further research is necessary.

The SEDAN (blood Sugar, Early infarct signs, hyper Dense cerebral artery sign, Age, NIH Stroke Scale)score[31]was reported[32] to show higher predictive value compared with other sICH risk scores, including MSS (Multicenter Stroke Survey)[33], HAT (Hemorrhage After Thrombolysis)[34], GRASPS (Glucose at presentation, Race [Asian],Age, Sex [male], systolic blood Pressure at presentation, and Severity of stroke at presentation [NIH Stroke Scale])[12], SITS(Safe Implementation of Thrombolysis in Stroke)[35], and SPAN (Stroke Prognostication using Age and NIH Stroke Scale)-100positive index[36]. Most risk factors of the SEDAN score, such as blood sugar, age and NIHSS, were found in this study; however, early infarct and hyper dense cerebral artery signs were not independent sICH risk factors as shown above. Future larger sample studies are required to confirm these findings.

Leukoaraiosis was shown to be a risk factor for sICH in acute ischemic stroke patients treated with IV r-tPA[37]. However, this aspect was not evaluated in this study. Further studies are required to confirm the above risk factors to guide clinical practice.

Nonetheless, risk factors for sICH should not be regarded as contraindications for IV r-tPA therapy in acute ischemic stroke.

There were some limitations in the current study.

Firstly, most participating hospitals in TIMS-China were in urban areas. Urban hospitals mainly accept urban patients and little rural patients, which may comply with patients choice bias.

Secondly, TIMS-China recruited patients from May 2007 to April 2012, and this long period may increase confounding factors. During six years of period, patients’ basic medical circumstance is changing. For example, habits and customs (smoking, drink, sports, etc) and medical prevention may change, which may comply with bias. Thirdly, the sample size was relatively small, and the obtained findings should be verified in future larger sample studies.

## Conclusions

Cardioembolism, NIHSS on admission higher than 20,serum glucose on admission >9.0 mmol/L and age ≧70 years are risk factors for symptomatic intracranial hemorrhage in Chinese patients with acute ischemic stroke treated with recombinant tissue plasminogen activator.

## Supporting information

S1 File TRAIS-CHINA ethic approval-Chinese version.pdf. (PDF)

S2 File TRAIS-CHINA ethic approval-English version.doc. (DOC)

S1 Checklist PLOSOne_Clinical_Studies_Checklist-PONE-D-17-01680.doc. (DOC)
