# Supplementary material for: Predictors of post-thrombolysis symptomatic intracranial hemorrhage in Chinese patients with acute ischemic stroke
Source: PLoS One. 2017 Sep 18;12(9):e0184646. doi: 10.1371/journal.pone.0184646 (PMC5602541; doi:10.1371/journal.pone.0184646)
Supplement: S1 File — (PDF) [file pone.0184646.s001.pdf]

北京天坛医院临床研究伦理委员会批件

临床研究项目名称	急性缺血性卒中溶栓登记研究（TRAIS）
项目承担单位	首都医科大学附属北京天坛医院
项目组织实施单位	中华人民共和国卫生部
研究内容简介： TRAIS 试验为“十一五”国家科技支撑项目分课题（项目编号 2006BAI01A11）。为开放的、多中心、前瞻性、非随机的观察性研究，预计在中国有资质进行急性缺血性脑卒中静脉溶栓的50家研究中心展开登记研究。符合纳入标准的患者将被随访 3 个月。随访内容：症状性脑出血和死亡作为主要安全性结局指标；生活自理能力作为次要结局指标。研究结果将公开和不断更新。指导委员会每 6 个月评价一次所有研究结果，决定是否建议修改研究设计或由于安全原因终止研究。	
同意者签字：[illegible]	不同意者签字
评审意见 经伦理委员会审议，符合相关规定和要求，同意该项目实施。 首都医科大学附属北京天坛医院 医学伦理委员会	
负责人签字 王拥军印	日期 2007-1-10 首都医科大学附属北京天坛医院 医学伦理委员会
