# Supplementary material for: Predictors of post-thrombolysis symptomatic intracranial hemorrhage in Chinese patients with acute ischemic stroke
Source: PLoS One. 2017 Sep 18;12(9):e0184646. doi: 10.1371/journal.pone.0184646 (PMC5602541; doi:10.1371/journal.pone.0184646)
Supplement: S2 File — (DOC) [file pone.0184646.s002.doc]

**Beijing Tiantan Hospital clinical research ethic approval**

| Clinical research name | Thrombolysis Register of Acute Ischemic Stroke(TRAIS-China) |
| --- | --- |
| Clinical research carrier institution | Tiantan Hospital,Capital Medical University |
| Clinical research executor institution | Chinese Ministry of Health |
| Clinical research introduction:  TRAIS is a secondary program of Chinese national technology program(serial number:2006BAI01A11).It is a open, multicenter, prospective, none random observal research, and about 50 centers may be taken part in.Patients will be followup for 3 months.Symptomatic intracranial hemorrhage is main end point, and Life self-care ability is secondary end point.Results of the research will be opened and renewed continuously.The trial will be assessed every six months, and may be modified or be terminated for safe consideration. | |
| Agreement signature:  Zhijiang Wang,Zhigang Zhao,Xiaoming Sun,Baoguo Wang,Zenghui Huo,Hai Zhou,Xinghu Zhang | Disagreement signature: |
| Review conclusion: Aprroved! |  |
| Responsible person signature:Yongjun Wang | Date:Jan 10, 2007 |

Note:Thrombolysis Register of Acute Ischemic Stroke(TRAIS-China) had be renamed as Thrombolysis Implementation and Monitor of Acute Ischemic Stroke in China (TIMS-China)
